# Adaptive MPPT control for reliable transitions between grid connected and islanded operations in PV battery microgrids

**DOI:** 10.1038/s41598-026-38300-5

**Published:** 2026-02-06

**Authors:** U. Siddaraj, Udaykumar R. Yaragatti, Lakhshman Rao S. Paragonda, Swathi Tangi

**Affiliations:** 1 Manipal Institute of Technology, Manipal Academy of Higher Education, Manipal, India; 2 Department of Electrical and Electronics Engineering, National Institute of Technology Karnataka, Surathkal, 575025 India

**Keywords:** The microgrid, Solar photovoltaic (PV), ANN–PSO algorithm, PQ controller, Storage system, Droop controller, SDG 7 - Affordable and Clean Energy, SDG 9 - Industry, Innovation, and Infrastructure, Environmental sciences, Energy science and technology

## Abstract

To maximize photovoltaic (PV) energy extraction, this study proposes a novel hybrid maximum power point tracking (MPPT) method that combines artificial neural networks (ANNs) with particle swarm optimization (PSO). The ANN–PSO controller is integrated within a PV-battery microgrid system and enables efficient tracking of the maximum power output while minimizing oscillations. The MPPT unit operates alongside a droop-controlled inverter to coordinate the power flow between the PV array and battery energy storage system (BESS), supporting dynamic transitions between grid-connected and islanded modes. The controller monitors the current and voltage at the point of common coupling (PCC) to ensure reliable power delivery. By enhancing the accuracy of irradiance estimations, the ANN–PSO approach improves tracking responsiveness and overall system efficiency. The proposed strategy is benchmarked against traditional and intelligent MPPT techniques, including Perturb & Observe, ANFIS-PSO, and PSO-SMC, under varying irradiance and load conditions. The performance is validated through detailed MATLAB/Simulink simulations. The results demonstrate superior tracking performance and faster, more stable microgrid operation, highlighting the controller’s potential for efficient renewable energy integration. This work supports the advancement of intelligent, autonomous energy systems and contributes to the development of resilient, grid-interactive solar microgrids. This research supports SDG 7: Affordable and Clean Energy by enhancing the efficiency and integration of solar energy systems, and it aligns with SDG 9: Industry, Innovation, and Infrastructure through the development of advanced control strategies for smart power grids.

## Introduction

The increasing energy demand, coupled with growing concerns about environmental degradation, has prompted electric power experts to explore sustainable methods of power generation^1^. Distributed generation (DG) from renewable sources, particularly solar energy, is recognized as a practical solution to reduce reliance on conventional power generation while improving the reliability and quality of power systems. Photovoltaic (PV) power systems have emerged as one of the most promising renewable generation technologies, benefiting from abundant solar resources and clean energy attributes^2^. The rapid advancement of PV technology and declining installation costs further contribute to its increasing deployment in power systems^3^.

However, owing to the inherent variability of solar energy and PV panels, the instantaneous power output of a PV system is heavily influenced by its operating environment, including solar irradiance and ambient temperature, leading to continual fluctuations in output power^4^. Consequently, battery storage systems are commonly integrated with PV systems to ensure consistent output power and mitigate variability^5^.

Given the dynamic nature of PV output power and load demand throughout the day, robust power management algorithms for the PV-battery system are essential for regulating power flow and swiftly adapting to changes, ensuring a balance between power generation and consumption^6^. Additionally, voltage stabilization for DC and AC buses is crucial to guarantee a consistent and reliable power supply, irrespective of system variations^7^. However, the conversion efficiency is inherently limited because of the nonlinear voltage/current characteristics of PV modules. Maximum power point trackers (MPPTs) ensure optimal PV energy generation by continuously tracking the global maximum power point^8^.

Various MPPT control strategies for PV converters have been extensively researched, as detailed in the literature^9^. Additionally, traditional MPPT methods such as Perturb & Observe (P&O), incremental climbing (INC), and hill climbing (HC) have been studied^10^. While the first two methods are relatively simple to implement in hardware, they suffer from substantial oscillations, resulting in power losses. Conversely, the INC method offers accuracy and flexibility amid fluctuating meteorological conditions, though it requires complex simulation and experimentation. Accurate analytical models, advanced metaheuristic MPPT techniques, and physical enhancement methods are proposed in these works, which considerably improve PV performance under partial shading conditions^11,12^. Their contributions lie in the exact modelling of PV parameters, robust MPA-based MPPT, and novel strategies of water-swirl power improvement, all put together to strengthen reliability and efficiency in shaded PV environments^13,14^. The integration of these techniques into grid-interactive PV systems and scalability analyses has not yet been fully addressed. However, under fluctuating solar irradiation, the effectiveness of the previously mentioned algorithms diminishes. An alternative MPPT tracker, such as an artificial neural network (ANN), addresses this limitation under variable weather conditions. ANN techniques rely on data and feature a multilayered form. However, the challenge with this approach lies in determining the architecture of the layers^15^. Fuzzy logic control (FLC) systems rely on rules and membership functions determined by probability to guide system operation, but they can be complex and sensitive to parameter changes^16,17^. Both ANN and FLC offer comprehensive methodologies for intelligence^18^. In contrast, the ANFIS technique combines rule-based inference with learned data for accurate demonstration while maintaining high precision^19^. The PSO technique is utilized to adjust the MPPT and PI controller parameters finely, effectively mitigating unanticipated losses in the model^20^.

Various contemporary AI approaches, including PSO, the firefly algorithm, ABC, and ACO, are applied to optimize problems^21,22^. Among these, PSO stands out for delivering excellent results with minimal user adjustments. PSO offers several advantages over these intelligent methods, including simplified analysis, straightforward execution, and cost-effective computational estimation. It enables quick and precise monitoring of PV power under diverse conditions. Thus, the efficiency of renewable PV power systems is a pressing issue in optimization problems and a rapidly growing research domain in academia. Existing MPPT methods, such as P&O, PSO, ANN, and hybrid intelligent controllers, have shown improvements in tracking efficiency, but they often fall short in achieving fast operation, low computational cost, and dynamic adaptability when deployed in real-time microgrid environments, especially during grid-connected-to-islanded transitions. Unlike prior studies that evaluate MPPT techniques in isolation or under simplified conditions, our work integrates a PSO-optimized ANN-based MPPT controller within a coordinated PQ/droop control framework for PV–battery hybrid microgrids. The proposed approach is specifically designed to support seamless grid transitions, maintain the PCC voltage and frequency stability, and reduce oscillations during dynamic conditions. We have now included a dedicated comparative discussion, supported by a newly added Table 1, which categorizes the major MPPT methods^23^ and highlights their working principles, key advantages, limitations, and ideal application scenarios.

**Table 1:** Summary of the major MPPT techniques used in solar PV systems.

MPPT Technique	Working principle	Key advantages	Limitations	Suitable applications
Perturb & observe (P&O)^4,9,10^	Periodic perturbation of voltage/current and observation of power change	Simple, low cost, easy to implement	Oscillations at MPP, mis-tracks during fast shading transitions; may converge to wrong peak in multi-peak PSC curves	Small standalone systems with mild irradiance variability
Incremental conductance (INC)^4,9,10^	Uses dI/dV = –I/V condition at MPP	Superior dynamic response; better tracking under moving clouds or non-uniform insolation	Moderate complexity, still susceptible to environmental changes	Rooftop PV, microgrids with moderate irradiance fluctuations
Constant voltage (CV)^4,10^	Operates at fixed percentage (~ 76%) of open-circuit voltage	Simple, requires minimal sensors	Large mismatch under temperature/irradiance drift;	Rural PV lighting systems
Fractional open-circuit voltage (FOCV)^4,10,24^	Samples Voc and estimates Vmpp as a fraction	Sensor-light, simple digital implementation	Requires periodic disconnection, affects energy harvesting	Off-grid or remote PV systems
Fuzzy logic control (FLC)^16,27^	Uses linguistic rules based on error and change of error	Handles nonlinearity well, adaptable	Requires heuristic rule design; performance depends on granularity of membership functions	PV systems with moderate unpredictability, hybrid microgrids
Artificial neural network (ANN)^8,15,27^	Trained on data to predict MPP	Fast, handles complex patterns, adaptive	Needs extensive training dataset; risk of model drift; higher computational footprint	Real-time systems with sufficient resources for smart inverters and large PV plants
Particle swarm optimization (PSO)^8,20^	Uses multi-agent swarm search to find global peak on multi-peak P–V curve	Avoids local minima, effective under partial shading	Slower , complex to implement in hardware	PV farms experiencing shadow patterns from buildings, trees, or trackers
ANFIS (adaptive neuro-fuzzy inference System)^19^	Combines ANN and fuzzy logic learning	High accuracy, intelligent decision-making	training complexity increases with input dimensions	Intelligent smart-grid applications where adaptive learning is needed
Sliding mode control (SMC)^4^	Forces PV voltage/current toward MPP manifold using high-speed switching	Robust to disturbances and uncertainties	Chattering effect, design complexity	PV in harsh or unstable environments

This study introduces a monitored algorithm to improve PV generation performance while effectively managing voltage and frequency in both off/on-grid modes. The proposed control method, which is based on ANN-PSO, is specifically designed to integrate photovoltaic systems with microgrids. A noteworthy aspect of this research is the novelty of the controllers, which focus on system coordination. By incorporating the suggested PSO-ANN MPPT control approach, which operates rapidly, the system maintains power balance on both sides of the converter while consistently regulating the DC-side voltage to keep the AC-side at the specified level via the inverter controller. The study of PV systems incorporates these MPPTs: P&O, PSO-SMC, ANFIS-PSO, and ANN-PSO. Moreover, microgrids offer notable advancements by ensuring precise power distribution and stable frequency and voltage during autonomous operations via appropriate controllers. Moreover, these control mechanisms seamlessly transition between modes, such as shifting P-Q control to V-f control during off-grid operation. The effectiveness of these control methods is substantiated by voltage and current measurements used to design controls in static reference frames. This approach simplifies and expedites controller development while eliminating the need for reference-frame transformations, thereby streamlining the overall system.

The uniqueness of this work lies in its coordinated control framework, rather than just the ANN–PSO optimization. The proposed controller integrates a PSO-initialized ANN-based MPPT with a hierarchical inverter control strategy for the PV system, comprising PQ control for the grid-connected mode and droop control for the islanded mode for the BESS, along with automatic resynchronization to ensure voltage and frequency continuity during transitions. To our knowledge, this integrated MPPT–inverter control scheme, which enables rapid MPP tracking and stable mode transitions, has not been previously reported.

The organization of the work is as follows: Section II presents a description of PV systems and proposes MPPT control with or without grid side control. Section III discusses the storage system’s transition from the grid to the coordinating droop/PQ controller. Section IV offers encouraging evidence of the studied control approaches. Finally, Section V concisely outlines the paper’s key findings.

### Description of proposed MPPT in photovoltaic systems

**Fig. 1:**
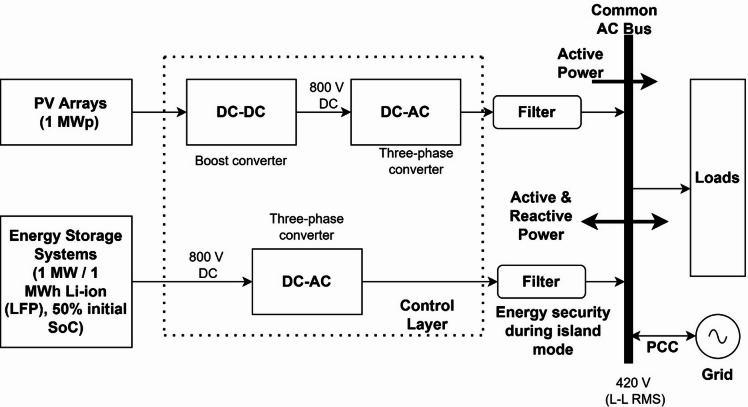
AC microgrid architecture.

A schematic representation of an AC microgrid is depicted in Fig. 1. A microgrid setup comprising a PV array with multiple panels connected to a boost converter with the proposed hybrid ANN– PSO-based MPPT and an inverter facilitating DC-to-AC power conversion is studied first. A unidirectional DC/AC converter is regulated by a voltage regulator connecting it to the AC bus. The PV system can operate in grid-connected modes^26,27^.

### Modeling and analysis of the solar PV

**Fig. 2:**
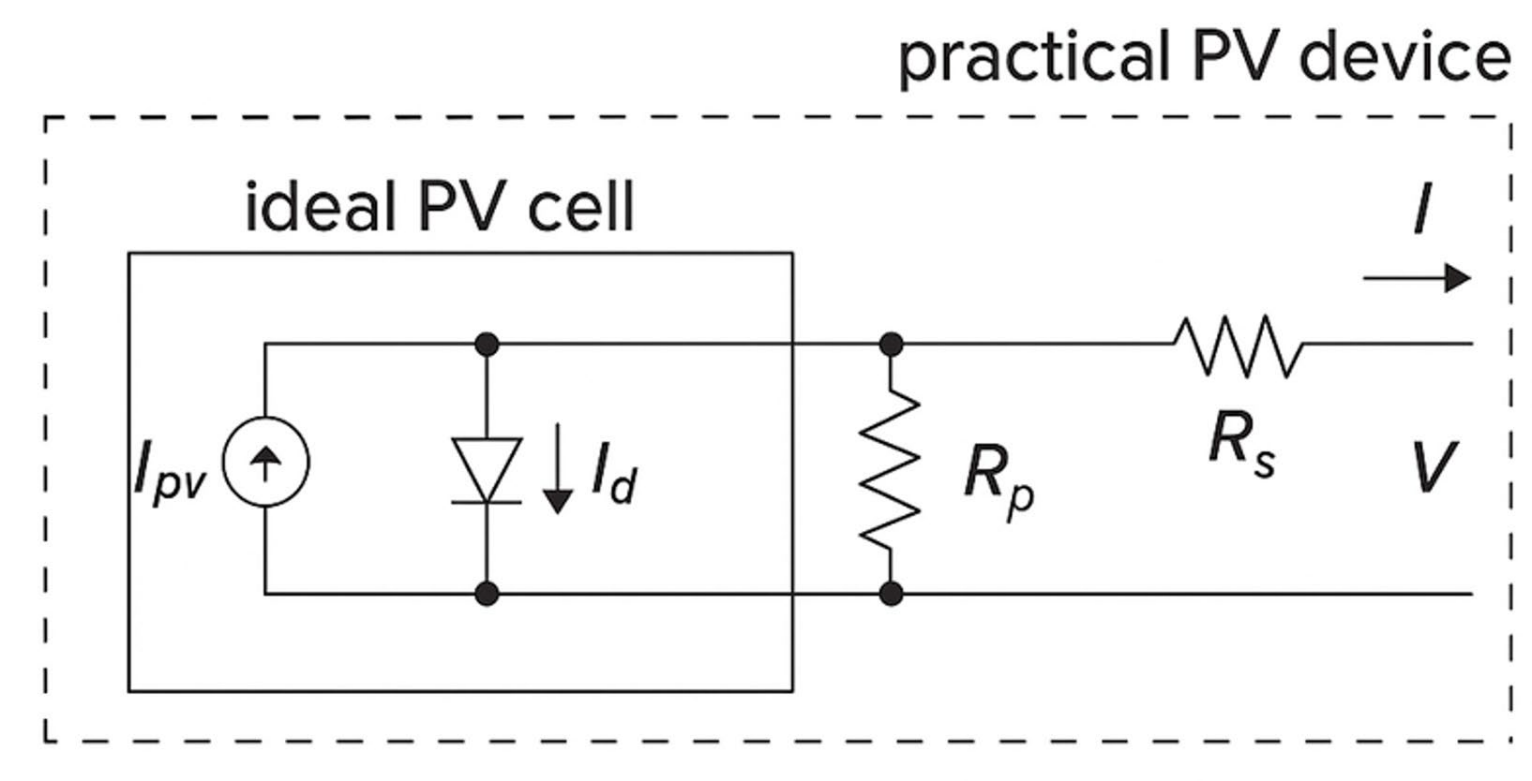
Practical photovoltaic cells.

The process begins with a model of a single solar cell, progresses to a PV module and culminates in an analysis of a whole PV array. Figure 2 shows that the PV cell has an output current of

1 $$I = I_{pv,cell} - \underbrace {{I_{o,cell} \left( {\exp \left( {\frac{qV}{{akT}}} \right) - 1} \right)}}_{{I_{d} }}$$

where ${I}_{o,cell}$ is the diode leakage current [A], ${I}_{d}$ is the diode Shockley equation. ${I}_{pv,cell}$: current produced by incident light; $a$: ideality constant; $T$: junction temperature of diode [K]; k: Boltzmann constant; and $q$: electron charge.

Equation (1), which represents the basic photovoltaic cell, does not fully capture the I-V characteristics of a real solar array. Additional factors must be incorporated into the fundamental equation to account for the features observed at the array terminals. This adjustment is necessary because practical modules consist of multiple interconnected photovoltaic cells^28^

2 $$I = I_{pv} - I_{o} \left( {\exp \left( {\frac{{V + IR_{s} }}{{V_{t} a}}} \right) - 1} \right) - \frac{{\left( {V + IR_{s} } \right)}}{{R_{p} }}$$

where ${I}_{pv}$ represents the module photovoltaic current, ${I}_{o}$ represents the module saturation current, and ${V}_{t}={N}_{s}kT/q$ represents the module thermal voltage when ${N}_{s}$ PV cells are added in series. The array equation is as follows:

3 $$I_{P} = N_{Par} I_{pv} - N_{Par} I_{o} \left( {\exp \left( {\frac{{V + IR_{s} \left( {\frac{{N_{Ser} }}{{N_{Par} }}} \right)}}{{V_{t} aN_{Ser} }}} \right) - 1} \right) - \left( {\frac{{V + IR_{s} \left( {\frac{{N_{Ser} }}{{N_{Par} }}} \right)}}{{R_{P} \left( {\frac{{N_{Ser} }}{{N_{Par} }}} \right)}}} \right)$$

The cells are connected in series to increase the voltage in the module, whereas the cells are connected in parallel to increase the current. The photovoltaic current ${I}_{pv}={I}_{pv,cell}{N}_{p}$ and saturation current are ${I}_{o}={I}_{o,cell}{N}_{p}$, respectively. Additionally, the module cells are connected in parallel to a ${N}_{p}$. In Eq. (2), the parallel and series resistances ${R}_{p}$ and ${R}_{s}$ are accounted for in the module.

**Fig. 3:**
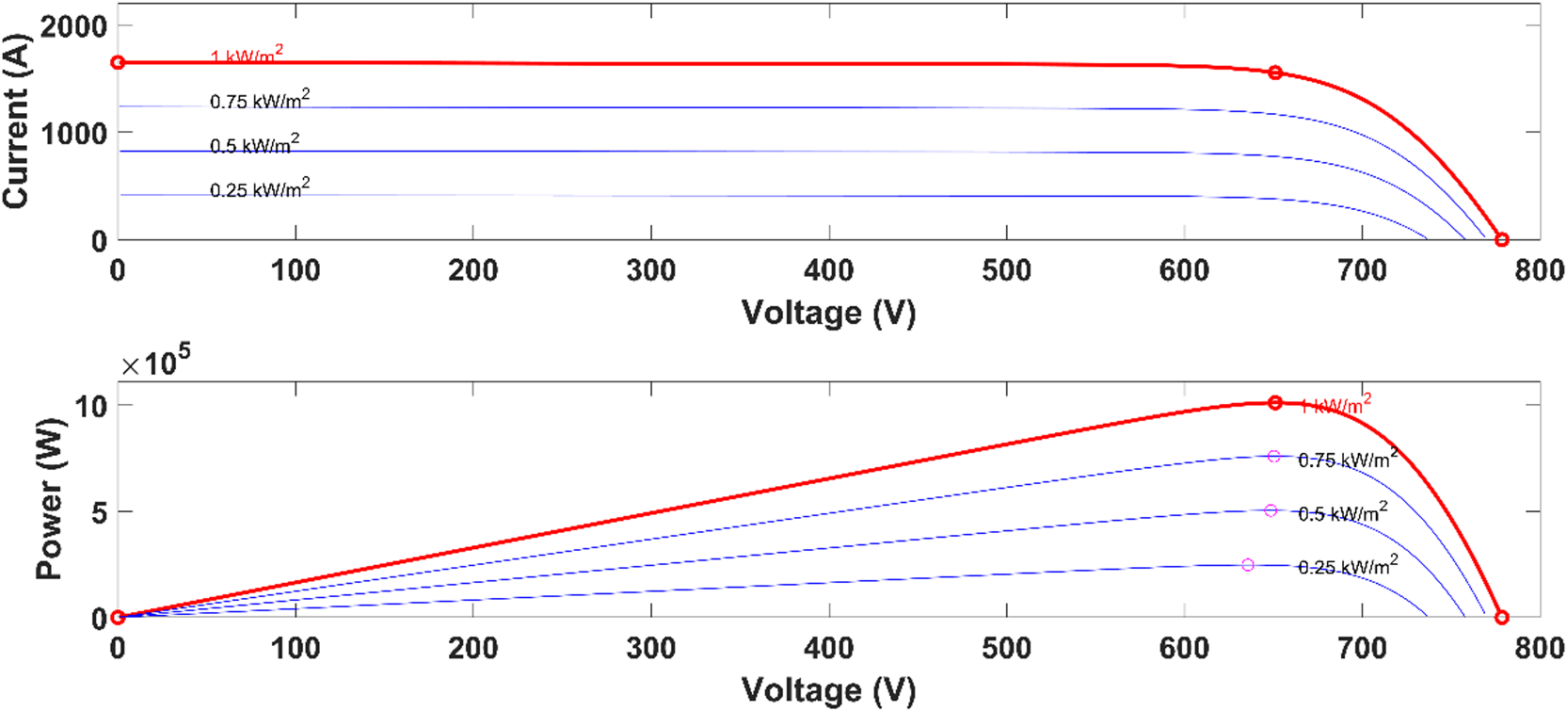
The adopted PV panel characteristics.

The adjusted photovoltaic model is developed and examined via the aforementioned equations and parameters with standard I‒V characteristics under varying insolation levels at 250 °C, as depicted in Fig. 3. The PV parameters of the panel being analysed are detailed in Table 2. The maximum power of the photovoltaic system at standard test conditions (1000 W/m^2^ and 25 ^°^C) of series panels 15 and parallel panels of 190 strings totaling 1000 kW is considered.

**Table 2:** PV panel parameters at 1000 w/M^2^ and 25 °C.

Parameters	Nominal value
${P}_{mpp}$	355.012
${V}_{mp}$	43.4
${I}_{mp}$	8.18
${V}_{oc}$	51.9
${I}_{sc}$	8.68

**Fig. 4:**
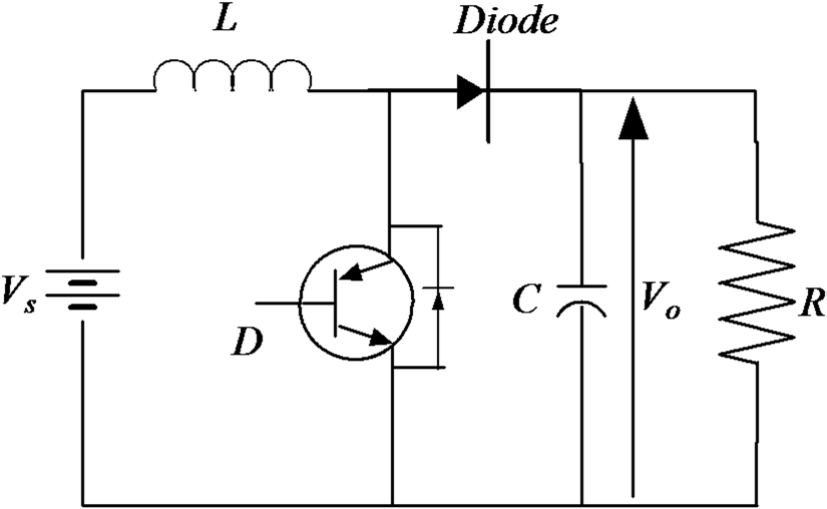
Boost converter coupled to a PV.

The maximum power point (MPP) of a photovoltaic (PV) system fluctuates with changes in the cell temperature and irradiance level. Figure 4 illustrates a converter connected to the PV system controlled by MPPT controllers. These controllers respond to variations in both radiation and temperature. Therefore, regardless of the conditions, the MPPT controllers employ various methods to continually correct the operational point of the MPP.

### PV MPPT PSO-ANN control

**Fig. 5:**
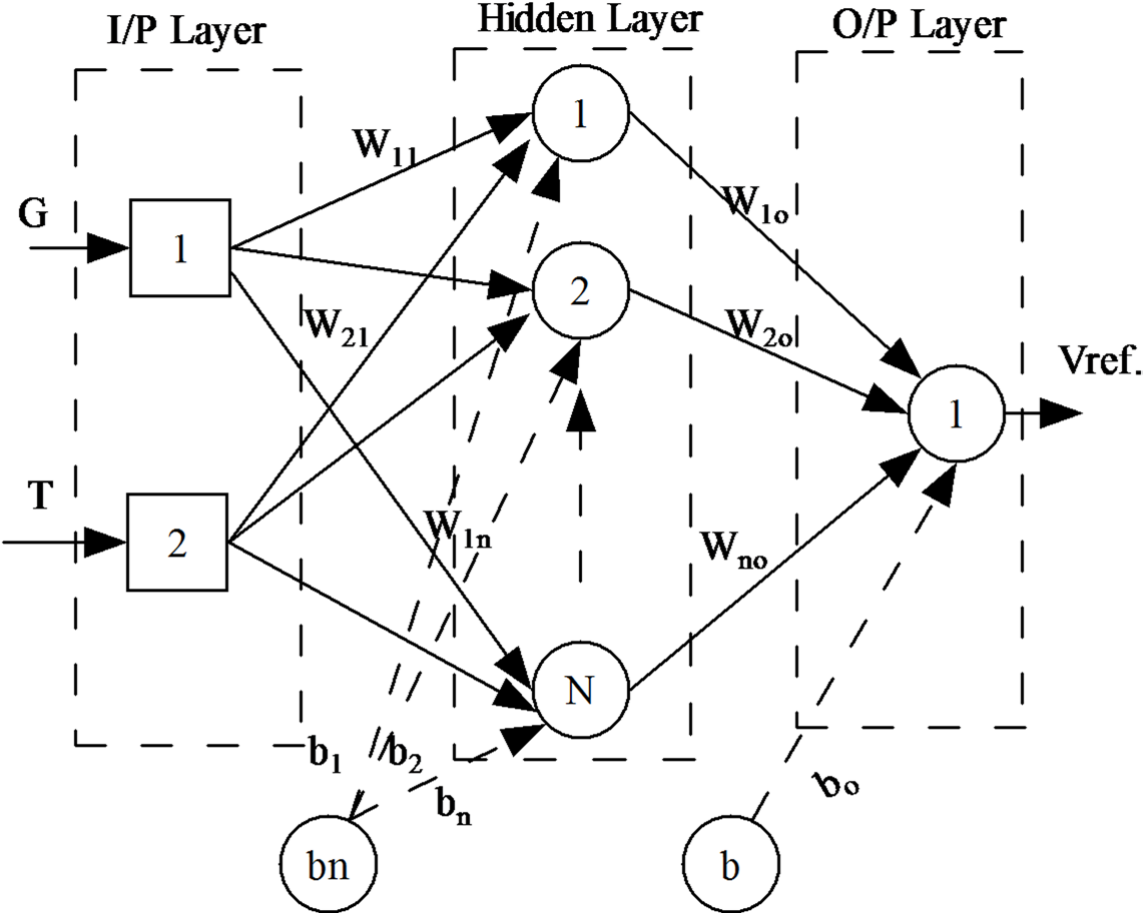
Block diagram of an ANN system.

An ANN approach, a form of distributed computing, needs reliable data to provide the most precise estimates of output functions. When applied to training data, this algorithm creates a nonlinear mapping between the input and the output nodes. The feedforward variant is the most frequently used because it requires less memory during implementation. Additionally, it is highly efficient when nonlinear systems such as a PV array are utilized. The input, hidden, and output layers make up the typical architecture of a multilayer feedforward ANN, as depicted in Fig. 5. Furthermore, all layer neurons are linked through the weights of their predecessors and the bias terms.

In most cases, the backpropagation (BP) technique is used to learn how a feedforward ANN works. Each node’s weights and bias terms are fine-tuned until the desired output is achieved via a complex gradient method. To minimize training errors, the value of the output layer is a forecast that comes as close as possible to the actual outcomes. The standard Eq. (4), which represents the MSE as the cost function, is as follows:

4 $$MSE = \frac{1}{n}\sum\limits_{i = 1}^{n} {\sum\limits_{j = 1}^{m} {[Y_{j} (i) - T_{j} (i)]^{2} } }$$

where $n$ represents the number of input data, $m$ represents the number of output signals, ${Y}_{j}(i)$ represents the real output, and ${T}_{j}(i)$ represents the target output. There are two main issues when designing a feedforward ANN: finding the.

The best topology of its structure (the number of hidden layers and units in these) and optimizing the initial weights of the training nodes.

Because of the trade-off between computing the amount of time and regression of the ANN nodes’ population that gives the best fit, it is crucial to calculate the appropriate number of hidden layers and units in each for the feedforward ANN design^25^. An overfitting regression occurs when the calculation time for an ANN model with many units in the hidden layers is too long. On the other hand, the computing time required to fit a linear regression model via an ANN will be minimal if the model contains too few units within its hidden layers. The thickness of a covert layer is often determined by trial and error. This approach, however, is insufficient since it takes so much time.

As shown in Eq. (5), this computational search evolves as a gradient descent proportional to the additional weight increment (ΔW),

5 $$w_{ji}^{l} (t) = w_{ji}^{l} (t - 1) + \Delta w_{ji}^{l} (t)$$

An update solution is generated in the first step of the $BP$ algorithm’s iteration. In the second, the MSE is calculated and updated with new weights via the above equations. This process is repeated until the ANN model’s training weights are optimal. This method fails to identify the training weights optimally because it primarily depends on the magnitude of $\Delta w$. If large, this can result in accelerated training and a significantly varying study of the error surface, leading to a nonconvergent optimized solution. If $\Delta w$ is small, however, training may be protracted, and the error surface may be examined seamlessly, leading to the training process being terminated before the global minimum error is discovered. According to this theory, the initial weights substantially affect how nearly an ANN model approaches the truth.

This process continues until the best training weights for the ANN model have been found. Several studies, for example, have shown that this approach fails to determine the optimized training weights since it largely relies on the size of $\Delta w$. A nonconverted optimized solution may result from rapid training and substantial fluctuations in the study of the error surface if $\Delta w$ is large. However, if $\Delta w$ is low, training will be sluggish, and the researcher may encounter fluctuations in the error surface without ever reaching a global minimum. In this context, constructing an appropriate ANN model relies heavily on the anticipated starting weights.

PSO-based optimization of ANN weights works by considering each candidate set of ANN weights and biases as a particle in the swarm. In every iteration, each particle is evaluated by inputting its weights into the ANN and calculating the resultant MSE. The particle’s velocity and position, i.e., weight vector, are updated based on this fitness value using three components: inertia due to previous motion, cognitive term due to its own best performance, and social term due to global best performance. Through these iterative updates, the swarm converges toward the weight vector that yields the minimum MSE. This optimized vector is then assigned to the ANN to ensure better initialization and faster and more accurate MPPT learning.

The PSO algorithm’s procedure consists of four stages. In the initial iteration, the PSO optimizer looks around for an arbitrary particle value. We have resolved this particle value to maximize the available solution space for various optimizations. The second stage compares the highest fitness values derived for the same space before ${P}_{bi}$ and after ${P}_{li}$. In the final stage, ${G}_{bi}$ is computed by comparing the best local and global positions. The transformed coordinates are then stored for use in the next steps via the following equation:

6 $$V_{i}^{k + 1} = w \times V_{i}^{k} + r_{1} \times C_{1} \times (P_{bi} - X_{i}^{k} ) + r_{2} \times C_{2} \times (G_{bi} - X_{i}^{k} )$$

7 $$X_{i}^{k + 1} = X_{i}^{k} + V_{i}^{k}$$

**Fig. 6:**
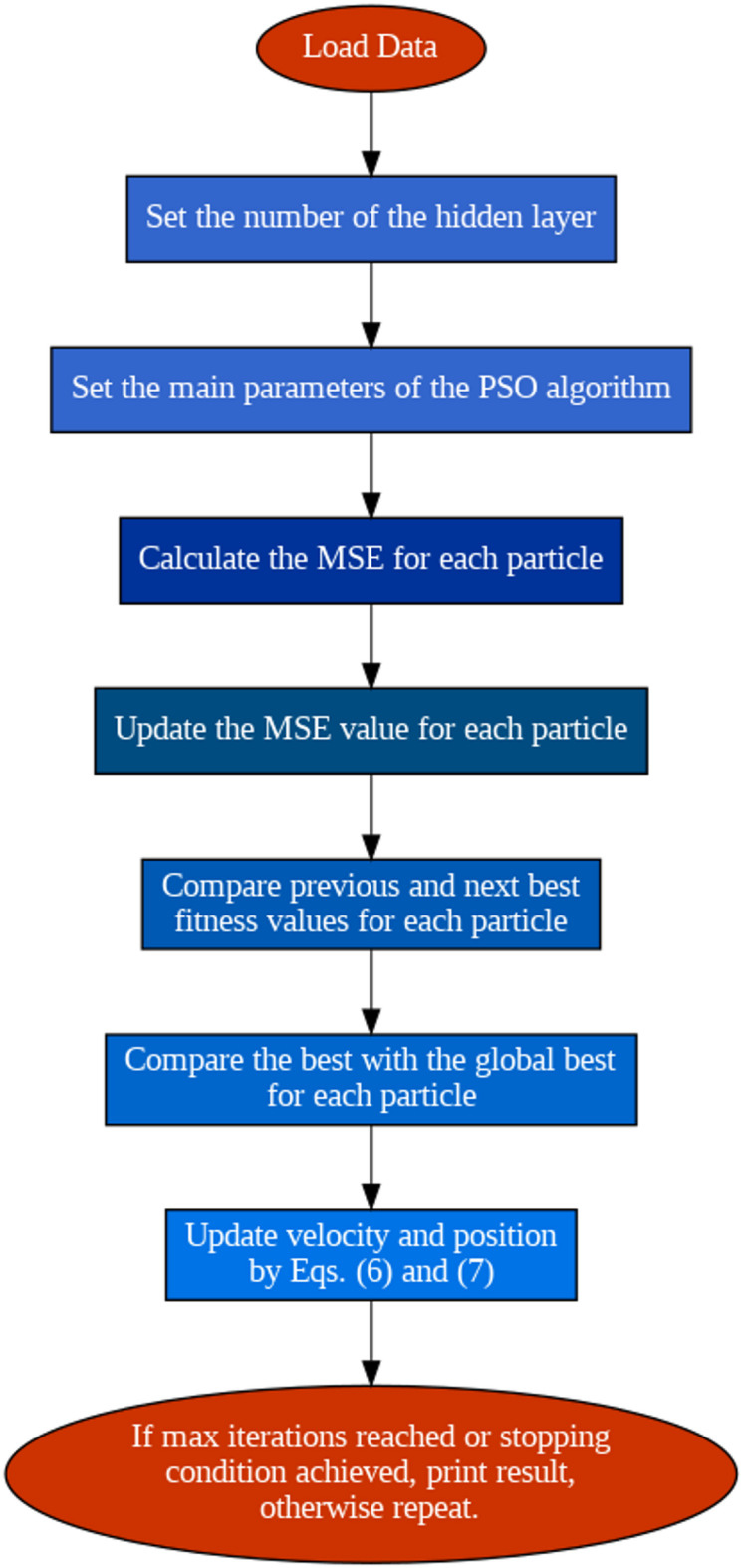
Illustration of the PSO-ANN training approach.

After a fitness check, the best particle is chosen and stored so that its motion may be fine-tuned with each repetition. These steps are continued indefinitely or until some stopping condition is satisfied. The system accuracy and processing time control requirements inform the suggested stopping condition and total number of iterations. Figure 6 depicts a flowchart of the development approach of the PSO–ANN algorithm.

Thus, a PSO ANN-based MPPT controller has been developed to generate $D$. As the output of the proposed model, the predicted voltage ${V}_{ref.}$ of the PV array at the MPP is calculated using the atmospheric conditions $G$ and $T$ as inputs. Following a PV operating simulator, an actual PV voltage ${V}_{act.}$ is measured under identical environmental conditions. The two voltages are then compared; thus, the error is passed to tune the controller to determine the converter’s $D$. The control signal is given by the following equation:

8 $$D = K_{P} (V_{act.} - V_{ref.} ) + \frac{{K_{I} }}{S}(V_{act.} - V_{ref.} )$$

Table 3 lists the parameters for training the data.

**Table 3:** Parameters of the PSO-ANN.

Parameters	Value
Population size	100
Iterations	250
Inertia weight	0.9
Personal learning coefficient (c1)	1.5
Global learning coefficient (c2)	2
No. of hidden layer	10
Data size	1000

A total of 1000 data samples were generated via random variations in solar irradiance (0–1000 W/m^2^) and temperature (15–35 °C). For each sample, the corresponding maximum power point (MPP) voltage, current, and power were computed via the PV module’s electrical parameters and temperature/irradiance coefficients.

**Fig. 7:**
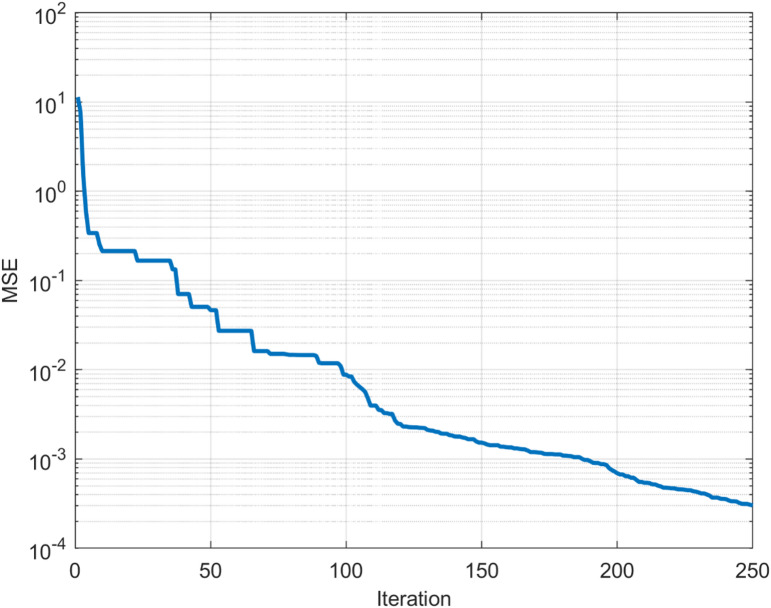
MSE vs Iteration using the PSO-NN.

The ANN was trained using irradiance and temperature as input features and the MPP voltage as the output target. The dataset was split into 700 training samples, 150 validation samples, and 150 test samples. The objective of a PSO-ANN algorithm is to determine the optimum initial weights for the model. To achieve this, the PSO algorithm and ANN technique are coupled. After running this hybrid algorithm, the optimum weights and biases are determined. The neural network model is trained after the optimal initial weights are selected. Thus, a lower MSE for iteration, approximately 0.0014 and 250, is shown in Fig. 7.

### Inverter control

The DC/AC inverter balances power through the inverter PQ control method. The DC voltage is maintained at a consistently adequate level for effective control by the DC voltage regulator controller^29^, regardless of the actual power output from the PV system.

**Fig. 8:**
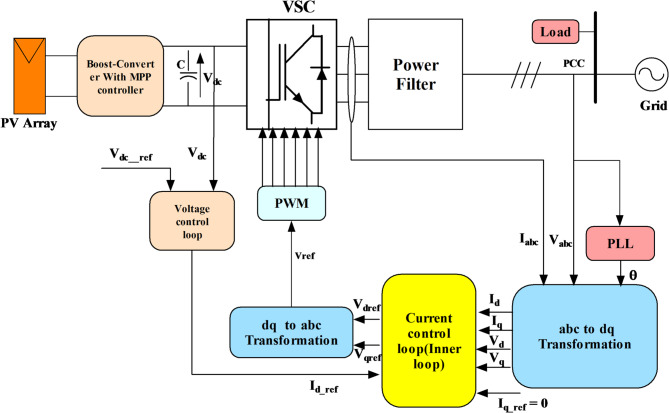
Block diagram of a three-phase PV inverter controller.

The main objectives of the converter controller involve regulating the voltage of the DC link within predetermined boundaries and transferring the active power from the distributed generation (DG) to the utility through the power electronic converters. Figure 8 depicts the control method. The DC voltage regulator task governs the voltage of the DC link and reference voltage to acquire the current reference. Thus, this reference current, along with the measured current, is fed into the inner current controller loop to calculate the reference voltage, which is subsequently utilized by the pulse generator to generate pulses for the inverter switches. Inverter losses were neglected, and the inverter was modelled as an ideal DC–AC stage. The incorporation of realistic switching loss models, such as turn-on/turn-off energy, tail current effects, and PWM-related losses, will be addressed in future work to provide a more complete power conversion efficiency analysis.

Using the standard PI controllers in the DC voltage regulator and the current controllers gives the reference values:

9 $$\left. \begin{gathered} I_{d - ref} = (k_{pvpv} + \frac{{k_{iviv} }}{s})(V_{dc - ref} - v_{dc} ) \hfill \\ I_{q\_ref} = 0 \hfill \\ \end{gathered} \right\}$$

10 $$\left. \begin{gathered} V_{d} = (k_{pib} + \frac{{k_{iib} }}{s})(I_{dref} - I_{d} ) \hfill \\ + V_{d} - \omega L_{ff} I_{qref} \hfill \\ V_{q} = (k_{pib} + \frac{{k_{iib} }}{s})(I_{qref} - I_{q} ) \hfill \\ + V_{q} + \omega L_{ff} I_{dref} \hfill \\ \end{gathered} \right\}$$

where ${k}_{pvpv}$, ${k}_{iviv}$, ${k}_{pib}$ and ${k}_{iib}$ are the PI controller gains of the voltage and the inner current loop, respectively.

### BESS and PV system with grid or autonomous mode

The PV system and BESS incorporate a storage element obtained from the MATLAB SimPowerSystem library, with the battery being a crucial component due to unpredictable weather conditions. The governing equations of the battery during discharge/charge of the lithium-ion iron phosphate (LFP)^30^ are as follows:

11 $$V_{BAT} = V_{{_{0} }} - R.i - K\frac{Q}{{\left( {Q - {\mathrm{it}}} \right)}}({\text{it + t*}}) + A.\exp ( - B.it)$$

12 $$V_{BAT} = V_{{_{0} }} - R.i - K\frac{Q}{{\left( {{\text{it }} - 0.1Q} \right)}}i* - K\frac{Q}{{\left( {Q - {\mathrm{it}}} \right)}}.{\mathrm{it}} + A\exp ( - B.it)$$

where ${V}_{o}$ is the constant of the battery voltage (V), $Q$ represents the battery capacity (Ah), ${V}_{BAT}$ denotes the voltage battery (V), $Q$ represents the constant of polarization (V/Ah), $R$ signifies the internal resistance (Ω), $i$ represents the battery current (A), A denotes the exponential zone amplitude (V), ${i}^{*}$ indicates the filtered current (A), and $B$ represents the inverse time constant of the exponential zone (Ah).

**Fig. 9:**
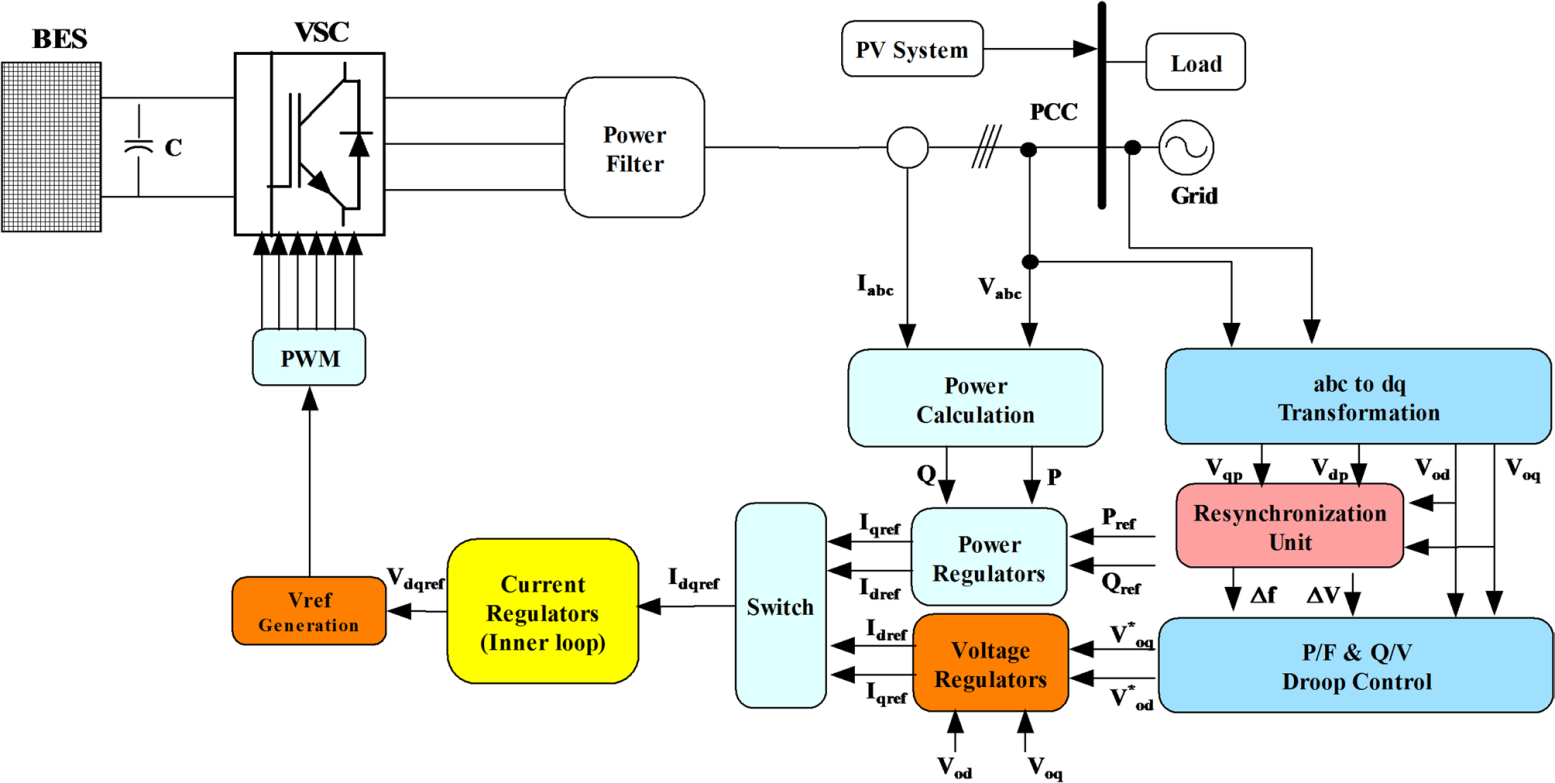
The storage system inverter controller.

In microgrid scenarios, dynamic load fluctuations and variable solar power generation necessitate the incorporation of a storage system. With a capacity of 1 MWh/1 MW, 50% SoC and a lithium-ion battery system with a nominal DC voltage of 800 V serve as a buffer energy source to sustain sensitive load stabilization. In addition to the battery system, the microgrid setup comprises a coupling transformer, filter, and inverter components. Figure 9 depicts the controller of the BESS, which is responsible for providing stable operation in a seamless microgrid transition with the grid. To ensure the stability of system frequency/voltage stability while maintaining a balanced generation and load profile, this controller integrates PQ, a droop controller, a power/voltage regulator, inner current control methodologies, and a phase-locked loop (PLL) to align the parameters of the PV battery and grid. Below is an in-depth justification of the controller.

### Unit of resynchronization

Reconnecting the microgrid to the supply system poses a risk of significant inrush currents if it is out of phase with the distribution voltage. Conversely, when the distribution and microgrid voltages synchronize, the resynchronization approach minimizes blackout risk and enables smooth reconnection to the supply system. The standard PI regulators accomplish this by gradually aligning the microgrid’s voltage and frequency with those of the central grid.

**Fig. 10:**
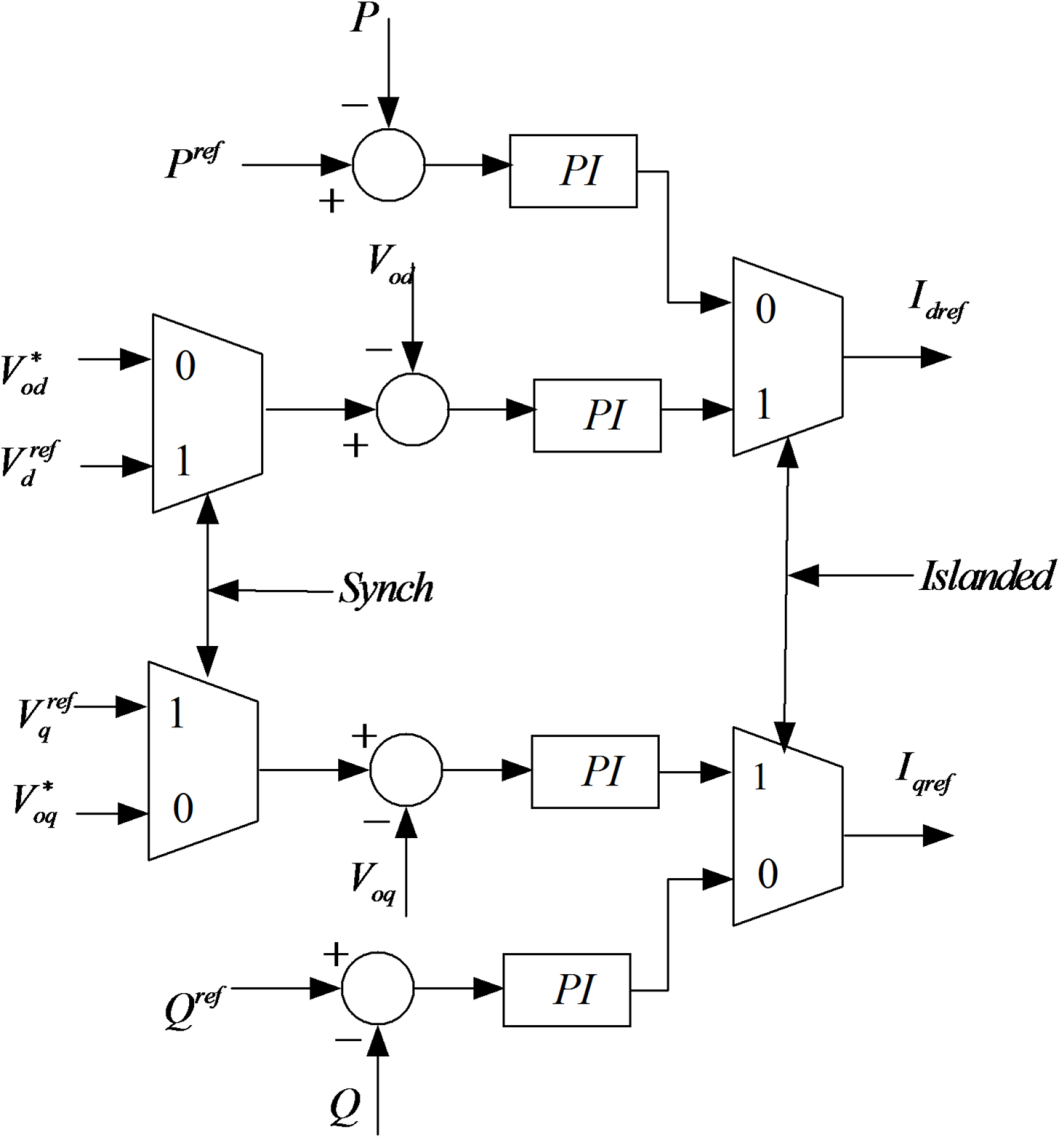
Controller of the transition modes.

**Fig. 11:**
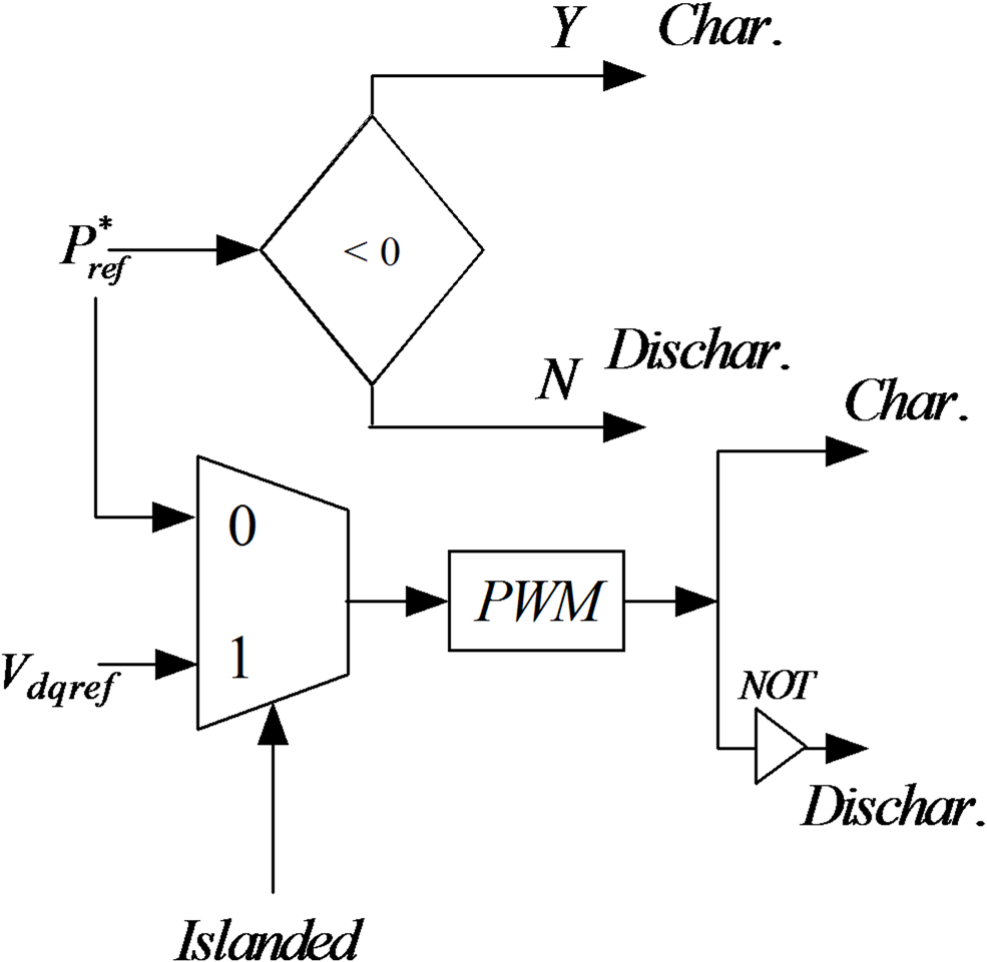
The control scheme of the battery.

During islanded mode, the signal “Sync” is “0” so that the controller has full control of the AC bus voltage by adjusting the references; for a smooth transition to grid-connected mode, “Sync” is set to “1” to synchronize the AC bus and grid-side voltages immediately before the breaker is closed. Hence, θ will be synchronized to follow the output angle of the PLL and the AC voltages after the breaker. Figure 10 shows the transition of modes and the control scheme of the battery, as shown in Fig. 11.

The resynchronization block calculates voltage and frequency deviations through a phase-locked loop (PLL)^31^. Adjustments are made to the primary droop to address these variations and accommodate changes in voltage or frequency.

As an energy buffer, it is necessary in PV systems for power balancing in autonomous mode.

The battery is connected to the DC bus and controlled by a bidirectional DC/AC converter in grid-connected mode. A single optimized control unit can manage both charging and discharging processes more seamlessly, and the power flow.

### Droop control

While operating in grid-forming mode, the microgrid’s battery energy storage system (BESS) manages both voltage and frequency. To ensure consistent active power output from the inverter and stable microgrid frequency, the BESS employs a droop control mechanism with a preset P/f parameter. The droop Q/V setting can also regulate the microgrid voltage at the point of the common coupling (PCC) bus. This dual-purpose droop control facilitates the adjustment of both actual and reactive power by regulating the voltage and frequency. The construction of a droop control equation and detailed information on a transmission line’s complex power are provided in^32^.

13 $$\left. \begin{gathered} P = \frac{{V_{1} V_{2} }}{X}\sin \delta \hfill \\ Q = - \frac{{V_{1} V_{2} }}{X}\cos \delta + \frac{{V_{1}^{2} }}{X} \hfill \\ \end{gathered} \right\}$$

*Sin*δ = δ and *Cos*δ = 1. Hence, Eq. (13) becomes

14 $$\delta = \frac{PX}{{V_{1} V_{2} }}$$

15 $$V_{2} - V_{1} \cong - \frac{XQ}{{V_{1} }}$$

The preceding equations demonstrate the utilization of real power for power angle regulation and reactive power for voltage management. Similarly, frequency control influences the power angle, impacting real power flow. Thus, the computation for the droop control coefficient is as follows:

16 $$\left. \begin{gathered} f = f_{s} + k_{pf} (P - P^{s} ) \hfill \\ V = V_{s} + k_{QV} (Q - Q^{s} ) \hfill \\ \end{gathered} \right\}$$

The $f/V$ are measured, and ${f}_{s}/{V}_{s}$ are reference values frequency/voltage, respectively; P and Q are measured; and ${P}_{s}$ and ${Q}_{s}$ are reference powers. The droop gains are ${k}_{pf}$ and ${k}_{QV}$.

### Power & voltage controller

During the operation of the microgrid, the voltage control loop is configured to compute ${I}_{d}$ and ${I}_{q}$ using the voltage reference, ${V}_{ref}$, produced by the droop controller, along with the detected $dq$ voltages. Conversely, the active and reactive power regulators come into play in grid mode. These regulators utilize measured and reference active/reactive power levels to generate reference currents ${I}_{dref}$ and ${I}_{qref}$. Equations (17) and (18) include the formulas governing the voltage and power regulators, including components of the $PI$ controller.

17 $$\left. \begin{gathered} I_{d} = (k_{pvb} + \frac{{k_{ivb} }}{s})(V_{ref} - V_{d} ) \hfill \\ I_{q} = (k_{pvb} + \frac{{k_{ivb} }}{s})(0 - V_{q} ) \hfill \\ \end{gathered} \right\}$$

18 $$\left. \begin{gathered} I_{dref} = (k_{ppb} + \frac{{k_{ipb} }}{s})(P - P_{ref} ) \hfill \\ I_{qref} = (k_{pqb} + \frac{{k_{iqb} }}{s})(Q - Q_{ref} ) \hfill \\ \end{gathered} \right\}$$

where ${k}_{pvb}$, ${k}_{ivb}$, ${k}_{ppb}$ and ${k}_{ipb}$ are the BESS voltage and the power controller $PI$ gains, respectively.

### Current controller

The current regulator receives currents via a switch, sourced either from the voltage controller (in off-grid mode) or the power controller (in grid mode). This regulator generates the $dq$ voltages necessary for producing pulses for the inverter. Notably, feedforward computation is employed by the regulators to ensure swift responses. The PI controllers ensure accurate tracking of current reference values, with current control acting as the inner loop of the inverter control system. The reference voltages, ${V}_{drref}$ and ${V}_{qrref}$, are derived by combining the output of the standard PI control with the dq voltage drop across the resistance and inductance ${R}_{ff}$ and ${L}_{ff}$. These reference voltages drive the pulse width modulation (PWM) mechanism, which generates the commands required for the inverter on the utility-connected inverter [33]. The governing equation of this process is as follows:

19 $$\left. \begin{gathered} V_{drref} = (k_{pib} + \frac{{k_{iib} }}{s})(I_{dref} - I_{d} ) \hfill \\ + V_{d} + R_{ff} I_{d} - \omega L_{ff} I_{q} \hfill \\ V_{qrref} = (k_{pib} + \frac{{k_{iib} }}{s})(I_{qref} - I_{q} ) \hfill \\ + V_{q} + R_{ff} I_{d} - \omega L_{ff} I_{q} \hfill \\ \end{gathered} \right\}$$

where ${k}_{pib}$, ${k}_{iib}$, ${k}_{pib}$ and ${k}_{iib}$ are the BESS current controller PI gains. ${V}_{dref}$ and ${V}_{qref}$ are transformed and scaled to produce a 3-phase reference voltage ${V}_{ref}$ to generate a PWM for the inverter.

## Results and discussion

The PV system with the studied MPPT control simulation is carried out in grid-following mode via the MATLAB/Simulink software package. The method presented in this section operates at the MPP point via four MPPT controls: P&O, PSO-SMC, PSO-ANFIS and the PSO-ANN MPPT controller. This article considers the utility mode with coordinated PQ control with different MPPTs. The PV system coupled with the PCC scenario is discussed in detail.

### Grid-connected mode

**Fig. 12:**
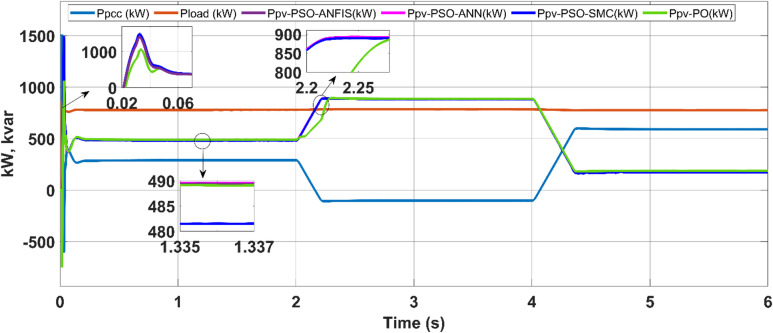
Power sharing of the PV with different MPPTs.

The study of grid-connected (PQ) controllers with intelligent MPPT control is summarized here. Table 4 shows the controller gain parameters. The proposed methods for the PV array work in MPPT mode, tracking the voltage reference estimated by the MPPT algorithm. The loads are connected to the bus. Depending on the quantity of power generated by the PV system and the grid’s capacity and demand, the power grid $({P}_{grid}$) absorbs or releases power to maintain a balance. Figure 12 shows a PV with MPPTs and grid power sharing and voltages at the system’s bus under PQ coordinated control.

**Table 4:** PV system parameters.

Symbol	Description	Nominal value
${k}_{pvpv}$	Proportional gain of current regulator	6
${k}_{ivpv}$	Integral gain of current regulator	30
${k}_{pib}$	Proportional gain of voltage regulator	0.2
${k}_{iib}$	Integral gain of voltage regulator	15
${F}_{SW}$	switching freq	2340

As shown in the power profile, prior to 2 s, the photovoltaic (PV) power output (P_PV_) is approximately 500 kW. During this interval, the utility grid supplies approximately 280 kW to meet the total load demand of approximately 780 kW, fulfilling the power balance condition P_load_ = P_PV_ + P_grid_. In this scenario, the PV system alone cannot satisfy the load, necessitating additional power support from the grid.

At approximately 2 s, a reduction in load demand occurs. Since the PV array continues to generate 500 kW while the load requirement drops, the excess power of approximately 100 kW is fed back into the grid. This reversal of power flow is indicated by the negative grid power (P_grid_ ≈ –100 kW), highlighting the transition from grid import to export mode. This demonstrates the system’s ability to inject surplus PV energy into the grid when generation exceeds consumption.

At 4 s, a sharp reduction in solar irradiance subsequently causes the PV output to drop to nearly 100 kW. In this situation, the grid resumes power delivery to supplement the load, compensating for the shortfall in PV generation. This dynamic exchange is effectively managed by the PQ coordinated control strategy, which ensures seamless power balancing by commanding the grid to act as an energy buffer, absorbing or supplying power as needed on the basis of instantaneous generation and demand profiles.

**Fig. 13:**
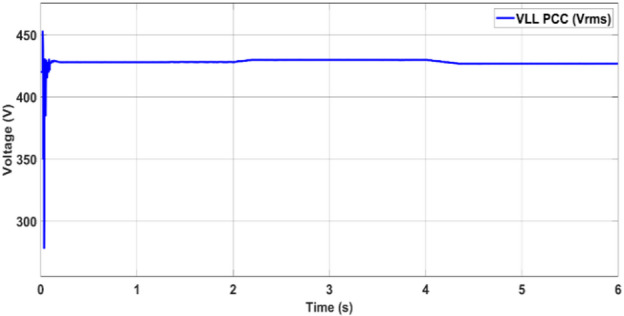
PCC voltage.

During the observed dynamic operating conditions, the point of common coupling (PCC) voltage is effectively regulated to ensure stable voltage delivery across the connected loads. As illustrated in Fig. 13, the root-mean-square (RMS) voltage (V_rms_) remains consistently maintained at approximately 420 V, even during changes in the power flow direction and varying grid interactions. This voltage stability demonstrates the effectiveness of the coordinated control strategy in mitigating voltage disturbances during transients.

**Fig. 14:**
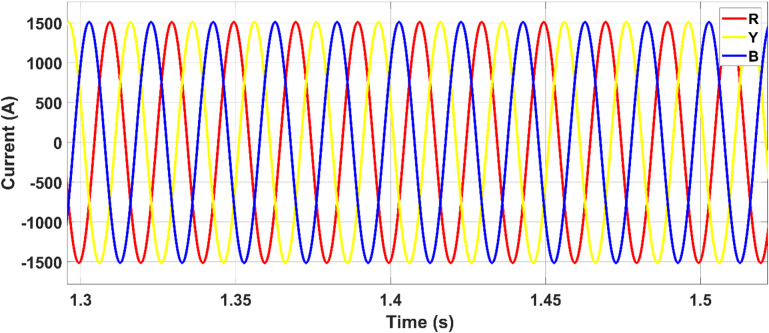
Load current.

Throughout the simulation period, the load demand remains nearly constant at approximately 800 kW. Consequently, the corresponding load current also remains stable, as shown in Fig. 14. This constant current indicates a steady power consumption profile from the load side, despite fluctuations in PV generation and grid contribution.

**Fig. 15:**
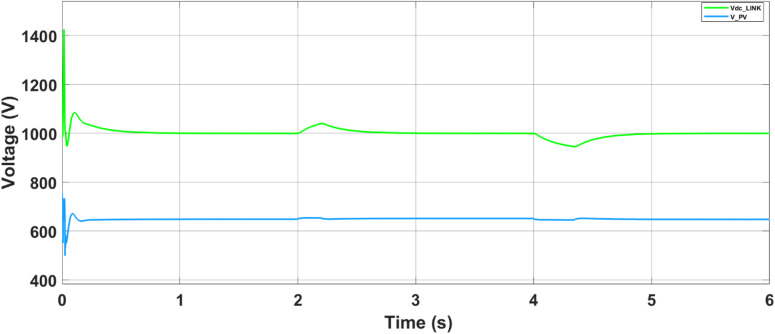
Output voltages of the PV (Vdc-link) and boost converter (Vpv).

The output voltage of the photovoltaic (PV) panel, referred to as the DC-link voltage (V_dc-link_), is regulated and boosted through the DC-DC converter, as shown in Fig. 15. This boosted voltage ensures optimal operation of the inverter and enables effective power injection into the microgrid.

**Fig. 16:**
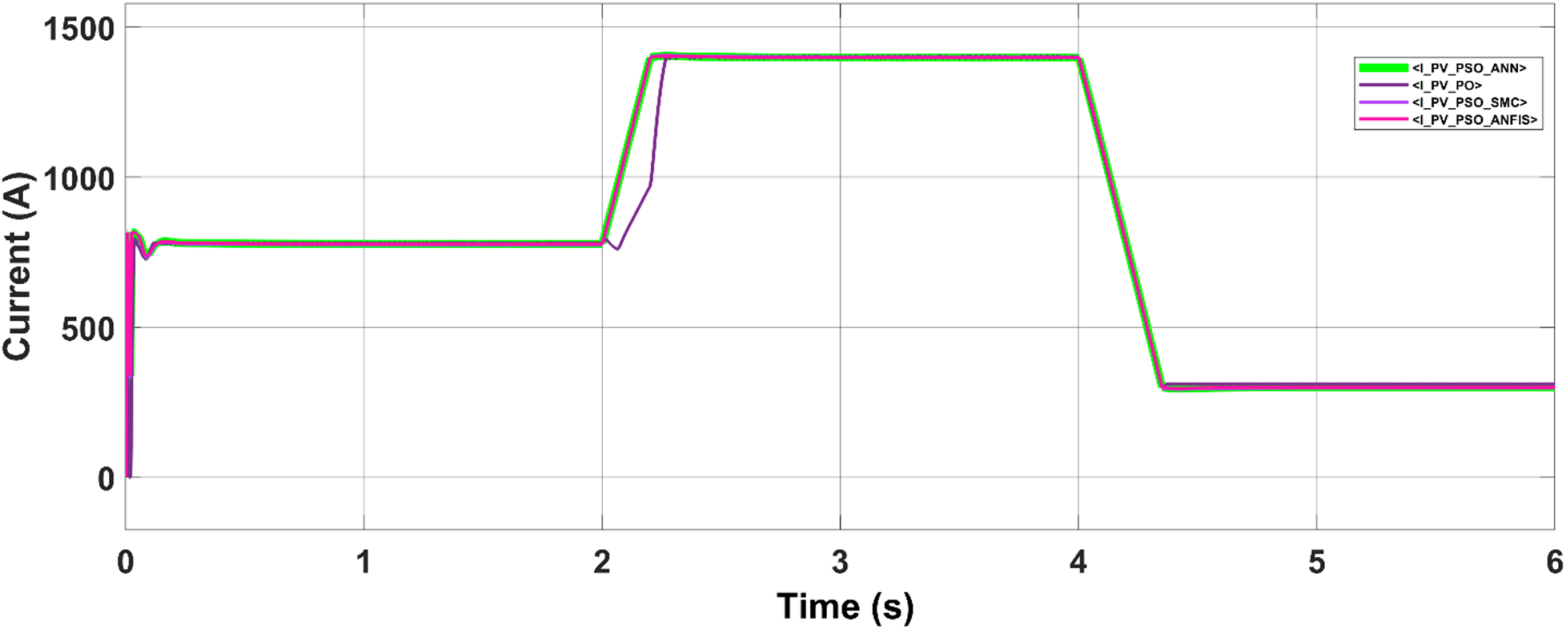
PV panel current with different irradiance values.

Since PV power production is inherently dependent on solar irradiance, any variability in irradiance directly influences the output current. Figure 16 shows that the load draws current from the PV source in proportion to the available solar power. In instances where PV generation is insufficient to meet the total demand, the grid seamlessly compensates by supplying the deficit power, thereby maintaining load‒generation balance.

**Fig. 17:**
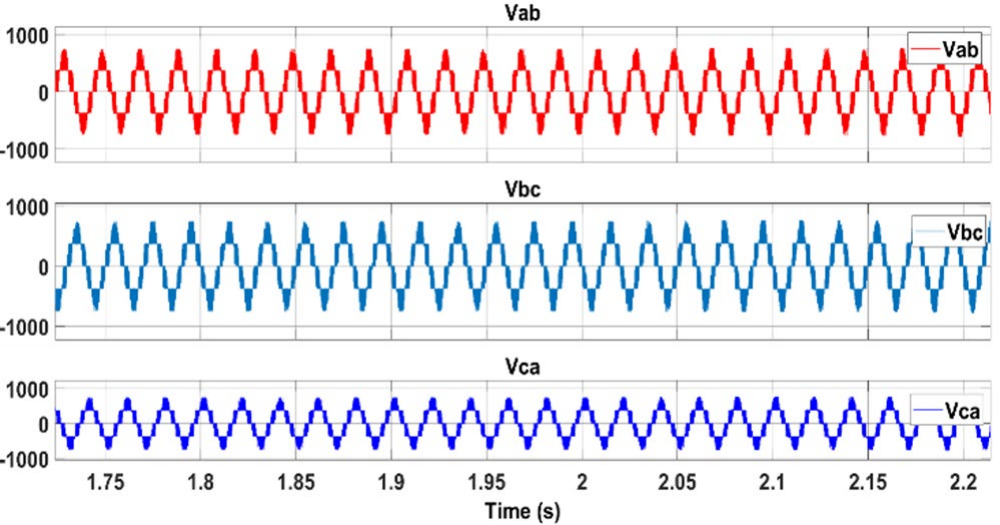
PV inverter output voltages.

Figure 17 depicts the output voltage waveform of the PV inverter prior to filtering by the LC stage. This waveform reflects the inverter’s raw switching output, which is subsequently smoothed to ensure high power quality at the PCC.

### Seamless transition from the grid-microgrid-grid mode

**Fig. 18:**
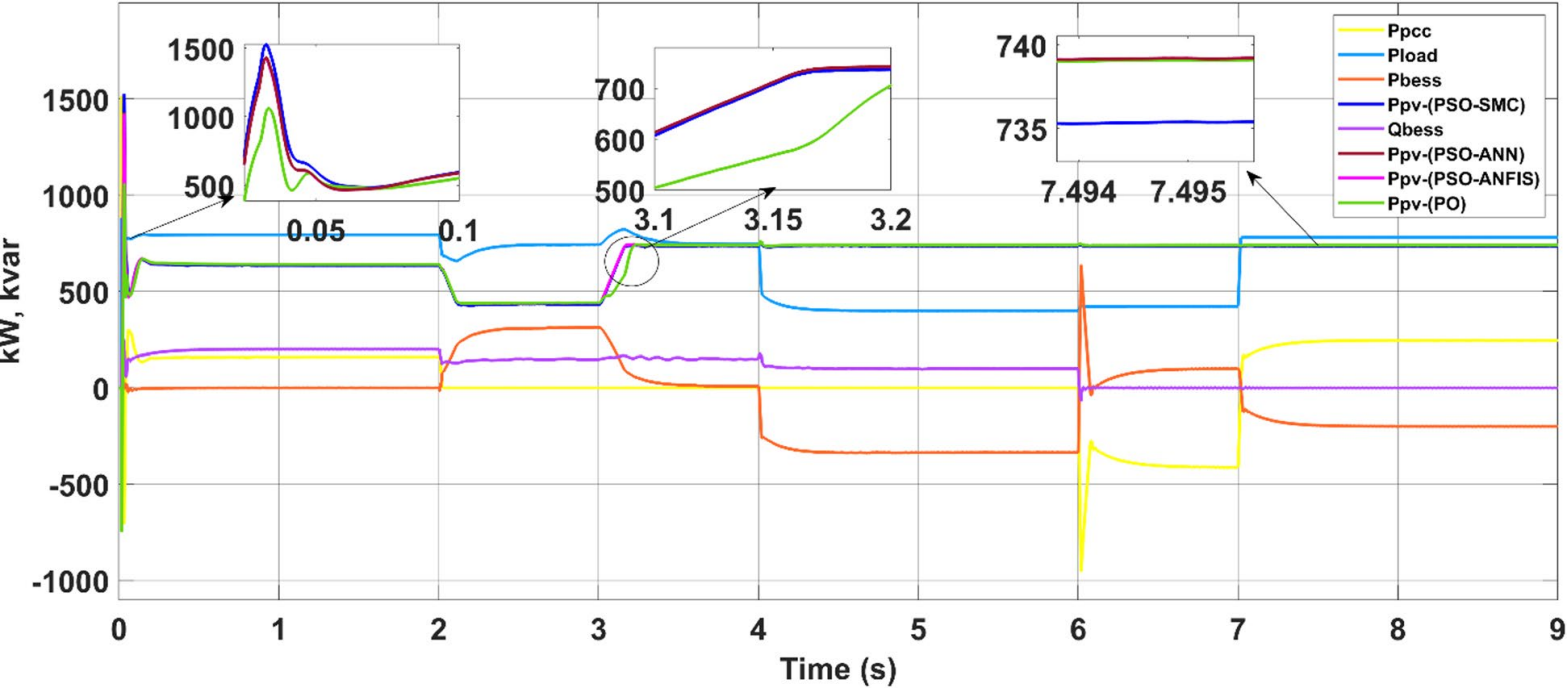
Load demand sharing by the microgrid.

A further case study highlights the dynamic adaptability of the implied control algorithms to shifts between grid and microgrid modes. Initially, operating as a conventional grid, the system intentionally transitions to an islanded microgrid setup by opening the tie switch after two seconds. Here, the microgrid relies solely on the battery energy storage system (BESS) connected to the point of common coupling (PCC) and the solar farm. The controller gain parameters are outlined in Table 5. The proposed maximum power point tracking (MPPT) controllers facilitate the coordinated injection of PQ power from the solar plant and BESS system during microgrid operation, as depicted in Fig. 18. These controllers ensure that the solar plant consistently produces active power at its maximum power point (MPP), guaranteeing reliable power generation in both grid-connected and microgrid modes while considering irradiance levels.

**Table 5:** Battery energy storage system data.

Symbol	Description	Nominal value
${k}_{pf}$	Frequency droop	0.5
${k}_{QV}$	Voltage droop	3
${k}_{ppb}$	Power regulator proportional gain	1.5
${k}_{ipb}$	Power regulator integral gain	15
${k}_{pib}$	Current regulator proportional gain	0.2
${k}_{iib}$	Current regulator integral gain	15
${k}_{pvb}$	Voltage regulator proportional gain	2
${k}_{ivb}$	Voltage regulator integral gain	25
${F}_{SW}$	PWM switching freq. Hz	2700

In microgrid mode, during the transition from the grid to the autonomous approach, the real power of the PV system is fed to the load, and the additional demand needed by the load is compensated by the BESS system with reactive compensation.

**Fig. 19:**
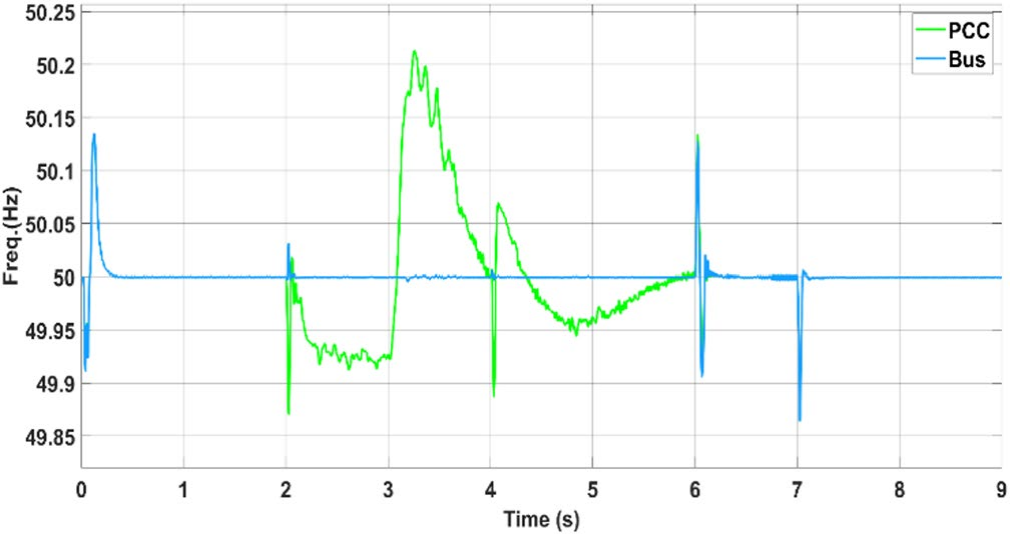
Frequencies of microgrids and PCCs.

**Fig. 20:**
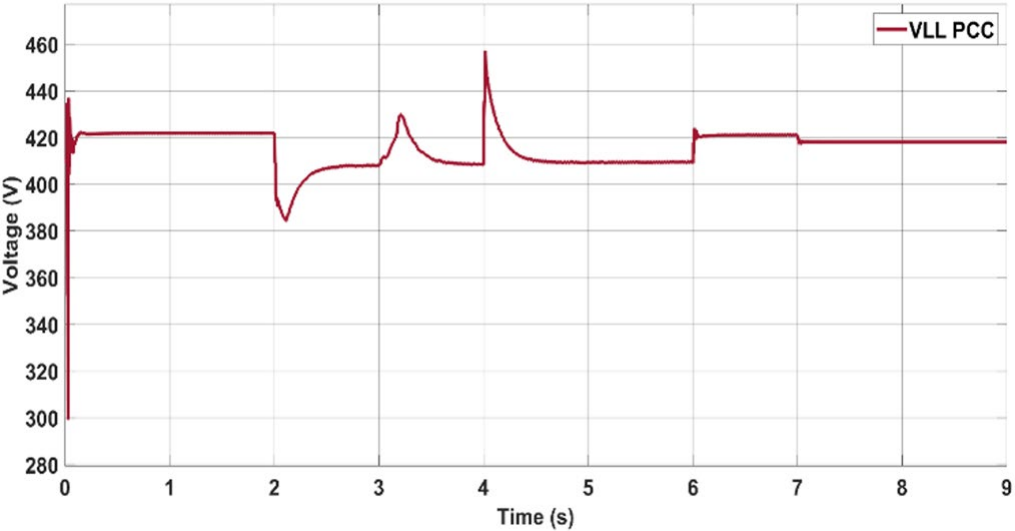
Voltage at the PCC.

In the first scenario, the PV–battery energy storage system (BESS) operates under normal grid-connected conditions. The PV array functions in MPPT mode, continuously tracking the optimal voltage reference (Vmpp) as determined by the ANN–PSO-based MPPT algorithm to ensure maximum power extraction. AC loads are connected to the common bus, and power management is governed by a coordinated control scheme. Depending on real-time power generation, consumption, and grid support (Pgrid), the battery either discharges (positive power) or charges (negative power) to maintain energy balance. The effectiveness of this coordination is illustrated in Fig. 18 (power sharing), Fig. 19 (frequency), and Fig. 20 (RMS voltage). Before 2 s, PV generation is approximately 650 kW, whereas the total AC load demand is approximately 780 kW. The 130 kW shortfall is supplied by the utility grid (Pgrid = 130 kW), keeping the battery in standby. This setup ensures stable voltage, frequency, and uninterrupted load support.

During the transition between grid-connected and islanded operations with a duration of 4 s, voltage (Fig. 19) and frequency (Fig. 20), the observed waveforms exhibit characteristics consistent with IEEE-1547 requirements. Specifically, the voltage profiles show no overshoot, discontinuity, or distortion spike during mode transfer, aligning with the IEEE-1547 allowable voltage deviation limits (≤ ± 10% voltage deviation during transients). Similarly, the frequency waveform remains stable without abrupt jumps or excessive rates of change, visually satisfying the IEEE-1547 frequency window (49.5–50.5 Hz).

**Fig. 21:**
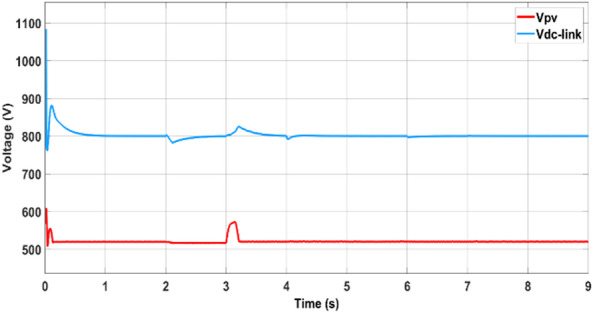
Output voltages of the PV and boost converter.

Following the initial power-sharing analysis, the output voltage behavior of the PV panel and the corresponding boosted voltage from the DC–DC converter are illustrated in Fig. 21. The PV panel output voltage dynamically adjusts on the basis of irradiance and temperature conditions, whereas the boost converter actively regulates the DC-link voltage to track the maximum power point as commanded by the ANN–PSO MPPT algorithm. This regulation ensures that the voltage is stepped up to a level suitable for interfacing with the inverter and maintaining stable operation of the AC-side loads. As shown in Fig. 21, the boost converter effectively stabilizes the output voltage during dynamic changes in solar input, reflecting the fast and accurate response of the MPPT controller.

**Fig. 22:**
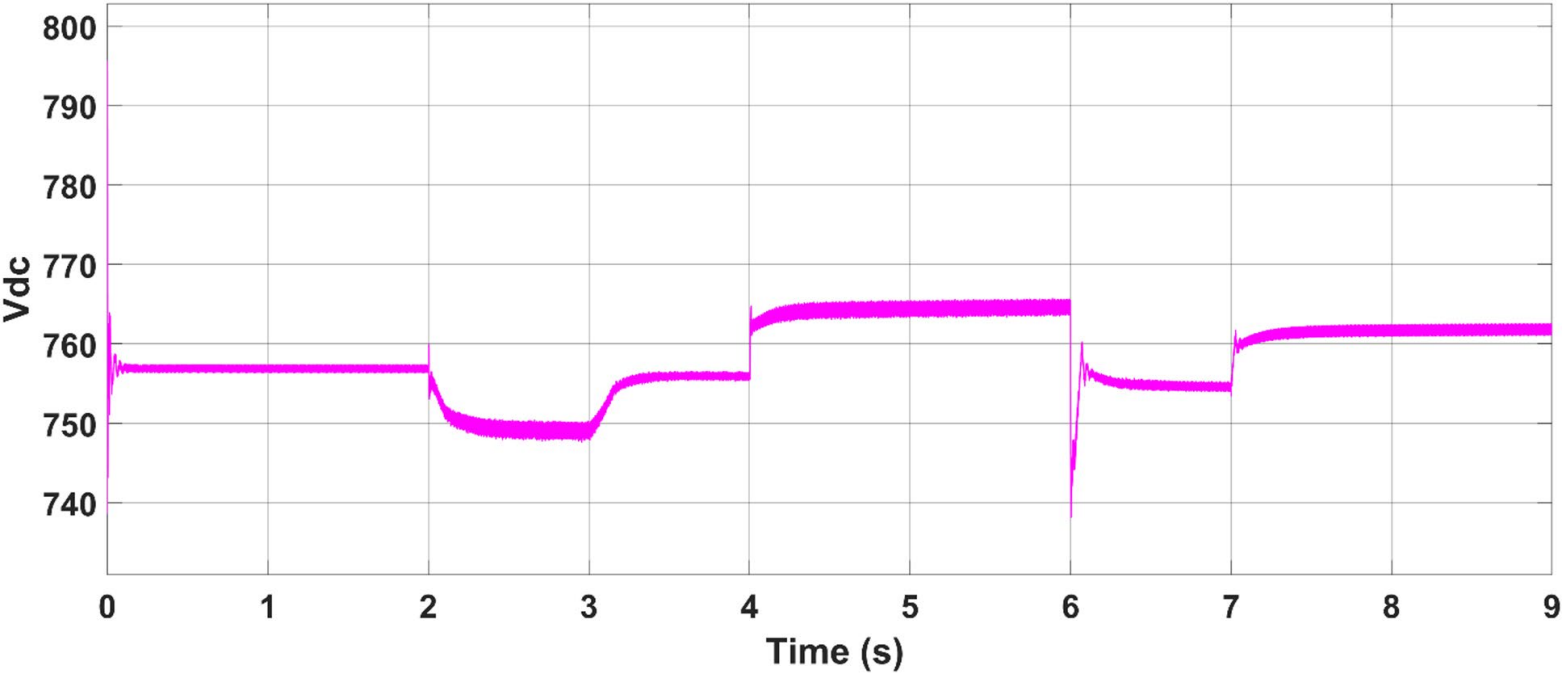
Battery storage DC link voltage.

**Fig. 23:**
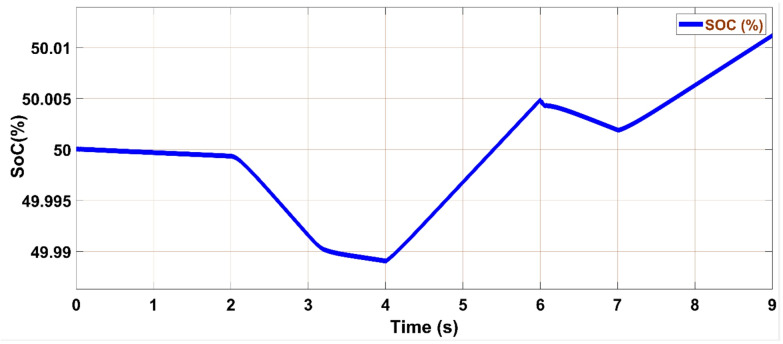
Storage SoC during load change.

The battery energy storage system (BESS) voltage, as depicted in Fig. 22, remains nearly constant throughout the entire simulation period, reflecting stable operation and effective voltage regulation. Figure 23 shows the battery’s active power profile. From 0 to 2 s, the battery remains inactive, as the PV and utility grid meet the load demand together, maintaining its state of charge (SoC) at 50%. At 2 s, upon grid disconnection, the battery begins supplying power to the load, with the power slope changing on the basis of the load current. At 4 s, as some loads are turned off and PV generation exceeds demand, the battery switches to charging mode. This charging continues until the grid is connected back at 6 s, showing the battery’s responsiveness to real-time power flow conditions. The results confirm that the proposed battery controller operates efficiently in both grid-connected and islanded modes, dynamically managing charge and discharge operations in response to fluctuating loads and PV generation.

As depicted in this figure, during any irradiation change, the proposed MPPT methods iterate until they reach the optimum voltage corresponding to the global MPP.

**Fig. 24:**
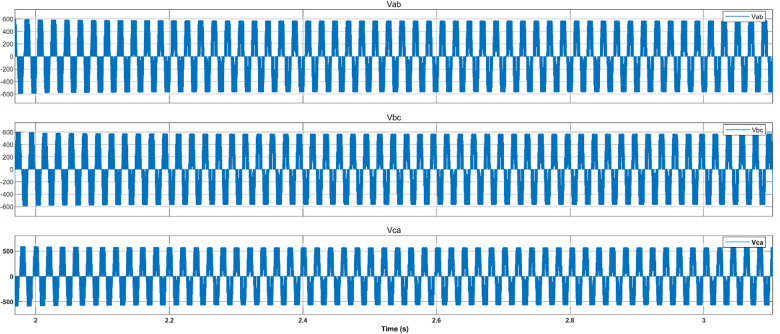
Battery energy storage inverter voltage.

**Fig. 25:**
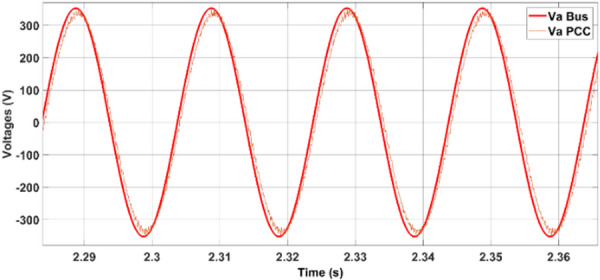
Islanding AC voltage.

The BESS inverter output voltages are shown in Fig. 24. Accidental acts of the PCC circuit breaker could result in disconnection of the PV-BESS. However, reconnection to the utility is usually arranged in advance, so the provider will have plenty of time to position the independent PV battery system for smooth reconnection before the breaker is closed. To avoid disruptions caused by breaker operation, the 3-ϕ voltage of the PCC must be synchronized with the grid side voltage. The following scenario compares the situations in which the voltages are synchronized by the proposed controller and those in which it is not before the breaker is closed. In Fig. 25, waveform distortion can be observed at the beginning of islanding (after 2 s) without voltage synchronization. In contrast, Fig. 25 presents the synchronizing process, which takes successfully at 6 s and avoids distortion. When the PV battery system is connected to the grid, disturbances are significantly reduced, ensuring that the bus loads receive a reliable power supply. A comparison of different MPPTs is presented in Table 6.

**Table 6:** Comparative performance of MPPT techniques under grid-connected and grid–microgrid transition modes.

mppt method	Operating mode	Mp (%)	Rise time (s)	Tracking efficiency (%)	Steady-state error (%)	Power loss (kW)
ANN–PSO	Grid-connected	90	0.012	97.80	2.20	17.60
ANN–PSO	Transition mode	90	0.026	97.80	2.20	17.60
PSO–ANFIS	Grid-connected	90	0.011	97.78	2.22	17.76
PSO–ANFIS	Transition mode	90	0.026	97.78	2.22	17.76
PSO–SMC	Grid-connected	95	0.010	97.74	2.26	18.08
PSO–SMC	Transition mode	95	0.025	97.74	2.26	18.08
P&O	Grid-connected	32	0.015	97.76	2.24	17.92
P&O	Transition mode	32	0.032	97.76	2.24	17.92

Table 6 reported MPPT performance indices (Mp, rise time, tracking efficiency, steady-state error, and power loss) for two different dynamic events that capture MPPT performance under both grid-connected and grid–microgrid transition mode conditions.

The results show that ANN–PSO consistently achieves faster settling, lower power loss, and comparable or superior tracking efficiency relative to PSO–ANFIS, PSO–SMC, and P&O across all dynamic scenarios. In particular, ANN–PSO reaches steady state earlier during load-change events (at ≈2.2 s and ≈3.15 s) while maintaining low steady-state error, confirming its suitability for fast and reliable MPPT under dynamic operating conditions.

This coordinated control mechanism allows the system to maintain power quality, support load fluctuations, and maximize the utilization of renewable energy, thereby enhancing grid reliability and resilience.

Summary of Key Findings:

- The proposed ANN–PSO method achieves the highest MPPT efficiency of 97.8%, outperforming PSO–SMC (**97.74**%) and P&O (**97.76**%).
- The steady-state power ripple in ANN–PSO is minimal (< 0.3%), indicating better tracking stability.
- The computational time is significantly lower in ANN–PSO than in other intelligent techniques, enhancing real-time feasibility.
- During low irradiance, ANN–PSO coordinates efficiently with the grid to maintain load-generation balance via PQ control and droop control.
- The PV inverter voltage and PCC voltage are stably maintained at a 420 V (LL) RMS throughout, even under a changing load and irradiance.

## Conclusion

This study presented a coordinated PQ/droop control framework integrated with a PSO-optimized artificial neural network (ANN)-based MPPT controller for dynamic photovoltaic (PV) power generation in a microgrid environment. The key findings and contributions are summarized below:

- The proposed ANN–PSO MPPT method achieved the highest tracking efficiency of 97.78%, with faster (0.036 s) and lower ripple (< 0.3%) than the PSO–SMC and conventional P&O methods.
- Under varying irradiance and load conditions (notably at t = 2.2 s in PQ control mode and 3 s in coordinated control mode), ANN–PSO demonstrated a superior dynamic response, settling faster than other techniques and maintaining power stability at the point of common coupling (PCC).
- The coordinated control strategy effectively managed mode transitions between grid-connected and islanded operations, utilizing a resynchronization unit for seamless grid reentry.
- Battery energy storage systems (BESSs), which are integrated with a droop controller, play crucial roles in frequency and voltage regulation, acting as energy buffers during power imbalances.
- In grid-following mode, the PQ controller ensures optimal power sharing among the PV, grid, and storage units. Excess PV energy was successfully diverted to the battery for later use, improving system self-sufficiency.
- This framework offers a scalable and intelligent solution for real-time PV integration in modern microgrids, enhancing stability, responsiveness, and energy utilization.

Future work will focus on implementing the proposed architecture in a hardware-in-the-loop (HIL) setup, followed by validation in a laboratory-scale PV–battery microgrid with real-world conditions.

## Data Availability

All the data generated or analysed during this study are included in this published article.
